# Health status of older cancer survivors—results of the PolSenior study

**DOI:** 10.1007/s11764-017-0672-6

**Published:** 2018-01-09

**Authors:** Joanna Sulicka, Agnieszka Pac, Monika Puzianowska-Kuźnicka, Tomasz Zdrojewski, Jerzy Chudek, Beata Tobiasz-Adamczyk, Małgorzata Mossakowska, Anna Skalska, Andrzej Więcek, Tomasz Grodzicki

**Affiliations:** 1 Department of Rheumatology and Balneology, Jagiellonian University Medical College, 31-531 Krakow, Poland; 2 Department of Epidemiology and Preventive Medicine, Jagiellonian University Medical College, 31-034 Krakow, Poland; 3 Department of Human Epigenetics, Mossakowski Medical Research Centre, PAS, 02-106 Warsaw, Poland; 4 Department of Geriatrics and Gerontology, Medical Centre of Postgraduate Education, 01-813 Warsaw, Poland; 5 Department of Preventive Medicine and Education, Medical University of Gdansk, 80-211 Gdansk, Poland; 6 Department of Pathophysiology, Department of Internal Medicine and Oncological Chemotherapy, School of Medicine in Katowice, Medical University of Silesia in Katowice, 40-752 Katowice, Poland; 7 International Institute of Molecular and Cell Biology, 00-109 Warsaw, Poland; 8 Department of Internal Medicine and Gerontology, Jagiellonian University Medical College, 31-531 Krakow, Poland; 9 Department of Nephrology, Transplantation and Internal Medicine, Medical University of Silesia in Katowice, 40-027 Katowice, Poland

**Keywords:** Cancer survivor, Elderly, Comorbidity, Health status, Falls

## Abstract

**Purpose:**

The purpose of this study is to characterize health status of older cancer survivors using data from the population-based PolSenior study.

**Methods:**

We compared cancer survivors and non-cancer subjects according to comorbidities, functional status, mental health, and sociodemographic factors.

**Results:**

There were 286 (5.8%) cancer survivors in a population of 4943 adults aged 65 years and older. The mean age of cancer survivors was 79.4 ± 8.2 years and the median time since cancer diagnosis was 8.5 years (Q1–Q3: 4–16 years). After adjustment for age, sex, education, marital status, and number of comorbidities, compared with a non-cancer population, cancer survivors were more likely to experience falls (OR = 1.38; 95% CI: 1.04–1.83), and to report poor health (OR = 1.49; 95%CI: 1.83–2.06), but cancer survivorship was not associated with impairments in instrumental activities of daily living (IADLs). Age and university education, but neither the time from cancer diagnosis nor the number of comorbidities, were associated with impairments in cancer survivors. Three or more chronic diseases were found in over 50% of cancer survivors and in 38% of the non-cancer population (*p* < 0.001).

**Conclusions:**

Cancer survivors over the age of 65 years have a higher prevalence of falls, are more likely to report poor health status, and have a higher number of chronic conditions than the non-cancer population, but they maintain independence in IADLs. Advanced age and elementary education are associated with increased occurrence of functional impairments in older cancer survivors

**Implications for cancer survivors:**

Older cancer survivors may require preventive services to reduce the risk of functional decline.

## Introduction

The prevalence of malignancy increases with aging, and around 60% of people diagnosed with cancer are over 65 years old. Cancer incidence rates in older adults in Poland in 2006 were 1818.5 per 100,000 in men and 923.1 per 100,000 in women, and cancer was diagnosed almost 2.5 times more often in adults over 65 years of age compared to individuals aged 45–64 years old [1]. Improved cancer diagnosis and care results in increasing numbers of survivors: There were over 32 million 5-year cancer survivors worldwide in 2012 (http://www.cdc.gov/cancer/international/statistics.html). The prevalence of cancer survivors in Europe is estimated to be 2% on average and 1% in Poland [2].

The toxicity of anticancer therapies in older individuals is added to age-associated alterations and an age-related increase in the prevalence of chronic diseases. Therefore, it has been recognized that older cancer survivors have a higher number of comorbidities: They are more likely to have one or more chronic conditions (42.1 vs 19.7%), and the age-adjusted prevalence of cardiovascular diseases is significantly higher in cancer survivors than in patients without a history of cancer (24.5 vs 22.9%). Patients who present with both cancer and concomitant diseases have an almost sixfold higher risk of psychological disorders (depression, anxiety, and adjustment disorders) than individuals without a history of cancer [3, 4].

The aim of this study was to characterize elderly cancer survivors (patients diagnosed with cancer, either currently undergoing treatment or currently free of cancer) in a representative Polish population-based sample of people aged 65 years and older, and to compare the occurrence of comorbidities, functional and cognitive abilities, and sociodemographic characteristics in relation to non-cancer subjects.

## Material and methods

### Study population

PolSenior is a population-based study conducted over the years 2007–2011 with a representative sample of the Polish population aged 65 years and older. The participants were recruited from all administrative regions in Poland using a three-stage stratified, proportional draw, with a response rate of 43%. The study was based on a standardized questionnaire, comprehensive geriatric assessment, and blood and urine sampling. The survey included data on socio-economic, functional, and cognitive status and concomitant diseases. The details of the design and recruitment criteria for the PolSenior survey, as well as descriptions of examination procedures and the structure of the study group have been reported previously [5].

#### Social characteristics

The measures included current marital status (classified in the present analyses as married or not married) and cohabitation status (living alone, cohabiting, and institutional), receiving any social benefits (yes/no), declared need for assistance in performing daily activities (yes/no), educational level (elementary, high school, and university), and living area (rural, urban with a population of at least 50,000, urban with a population of more than 50,000).

#### Self-reported health status

All participants were asked to assess their present general health using the 10-point visual analogue scale (VAS); in the analyses, a score of 0–4 was classified as “poor health.”

#### Cancer history

All participants were asked about their history of cancer, cancer type, age at diagnosis, and the status of cancer treatment (cured = treatment completed at least 5 years prior to the study; last treatment within 5 years prior to the study; currently undergoing clinical treatment). In the analyses, we use the term “cancer survivors” to define patients diagnosed with cancer undergoing treatment or cancer-free [2] (https://cancercontrol.cancer.gov/ocs/statistics/definitions.html). Answering “yes” to the question “Have you ever been diagnosed with cancer?” defined a person as a cancer survivor. In this article, the terms “cancer survivors” and “patients with self-reported cancer history” are used interchangeably.

#### Chronic conditions

The study considered cardiovascular diseases (including hypertension, coronary heart disease, myocardial infarction, and stroke), respiratory, digestive, endocrine, and metabolic diseases (including diabetes mellitus), blood diseases (anemia), kidney diseases, osteoporosis, and eye diseases.

#### Geriatric assessment

This included the occurrence of falls within 12 months prior to the study, the ability to perform instrumental activities of daily living (IADLs) measured using the Lawton IADL scale (no deficits in IADL defined as a score of 24/24), screening for depression using the short version of the Geriatric Depression Scale (GDS) (depression defined as a score ≥ 6/15), and cognitive impairment assessed using screening test: the Mini-Mental State Examination (MMSE) (< 24/30 points indicating impaired cognitive performance).

## Statistical analyses

To describe the data collected, mean values (for age) and median values for the first (Q1) and third (Q3) quartiles of the duration of disease were used. The cancer survivor group was compared with the group of respondents with no self-reported cancer history (non-cancer population) using the chi-squared test of independence, and the frequency of given conditions was presented. The relationship between self-reported cancer status and the presence of some limitations in functional activity, depression, dementia, need for support in everyday life, and falls, as well as poor self-rated health, was analyzed using multivariate logistic regression models. The set of possible covariates, including age (5-year groups, reference age 65–69), sex (ref. male), marital status (ref. not married), university education (ref. elementary education), and number of chronic diseases reported (none, 1, 2, or 3+), was used in all models. The results were presented as odds ratios (ORs) and 95% confidence intervals (95% CI). The next step of the analysis was to assess if there was any relation between the time from cancer diagnosis and the studied outcomes among respondents with a self-reported cancer history using multivariate logistic regression with the following covariates: age, sex, education, marital status, and number of comorbidities. For the purpose of this analysis, as a consequence of a number of missing data concerning the time of diagnosis and present status of the disease, analyses were performed with a smaller sample size. All analyses were performed in STATA 13 (StataCorp LLC, Texas, USA), and the statistical significance for all tests was 0.05.

## Results

### Characteristics of cancer survivors

There were 286 cancer survivors (5.8%) in a population of 4943 adults aged 65 years and older. The mean age of cancer survivors was 79.4 ± 8.2 years. In all, 72% of cancer survivors were 75 years and older and 44.8% were women. Nearly 60% of cancer survivors declared being cured of cancer; the other participants were either currently undergoing clinical treatment or had completed treatment less than 5 years prior to the study. The median time since cancer diagnosis was 8.5 years (Q1–Q3: 4–16) and median age at cancer diagnosis was 68 years (Q1–Q3: 60–76). Nearly two out of three cancer survivors (65.4%) were diagnosed with cancer over the age of 65 years, and 39.3% of cancer survivors were diagnosed with cancer 10 or more years prior to the study.

The level of education in cancer survivors was higher than in the non-cancer population, and they were more often urban dwellers, while marital and cohabitation status was comparable in both groups. Cancer survivors more often declared the use of social services in comparison to the non-cancer population. The characteristics of respondents with and without a history of cancer are presented in Table 1.

**Table 1:** Characteristics of cancer survivors and adults without a history of cancer from the PolSenior Study

	Self-reported cancer history			No reported cancer history			*P* value
	Total *n* = 286	Men *n* = 158	Women *n* = 128	Total *n* = 4657	Men *n* = 2394	Women *n* = 2263	
Age in years (SD)	79.4 (8.2)	80 (7.8)	78.6 (8.6)	79.3 (8.7)	79.4 (8.6)	79.2 (8.1)	NS
Age							*< 0.001* ^*a,c*^
65–74	28.3	23.4	34.4	34.7	34.0	35.4	
75–84	45.1	49.4	39.8	32.1	32.6	31.7	
> 85	26.6	27.2	25.8	33.2	33.4	32.9	
Male gender	55.2	–	–	51.4	**–**	**–**	NS
MMSE < 24 pts	24.8	23.1	35.6	32.2	30	32.9	*0.012* ^*a*^
IADL < 24 pts	49.8	49	50.8	52.2	50.2	54.2	NS
No. of chronic diseases							
0	9.5	10.8	7.9	16.4	20	12.6	*< 0.001* ^*a,b*^ *0.022* ^*c*^
1	22.1	25.3	18.1	22.8	23.9	21.6	
2	17.9	22.8	11.8	22.8	23.3	22.4	
3	50.5	41.1	62.2	38.0	32.9	43.4	
Education							*< 0.001* ^*a*^
Elementary	45.1	39.5	52.0	59	50.8	68.7	
High school	40.0	40.8	39.0	33	38.9	26.7	
University	14.9	19.7	8.9	8	10.3	5.5	
Marital status							NS
Not married	49.4	32.9	69.6	50.6	29.9	72.6	
Married	50.6	67.1	30.4	49.4	70.1	27.4	
Living alone							NS
Yes	20.5	14	28.5	21.5	14.5	28.8	
No	79.5	86	71.5	78.5	85.4	71.2	
Living in long-term facility							NS
Yes	1.5	1.4	1.7	1.1	1.1	1.1	
No	98.5	98.6	98.3	98.9	98.9	98.9	
Social services							*0.001* ^*a,c*^
Yes	6.2	6.6	5.7	2.8	1.9	3.6	
No	93.8	93.4	94.3	97.2	98.5	96.4	
Area of residence							*< 0.001* ^*a,b,c*^
Rural	28.0	29.8	25.8	40.6	39.2	41.9	
Urban < 50,000	21.0	19.6	22.7	25.7	26.2	25.1	
Urban > 50,000	51.0	50.6	51.6	33.8	34.5	33	

Note values indicate percentages except for age in years

^a^Cancer survivors vs. non-cancer population

^b^Women cancer survivors vs. non-cancer women

^c^Men cancer survivors vs. non-cancer men; *NS* not significant

**Fig. 1:**
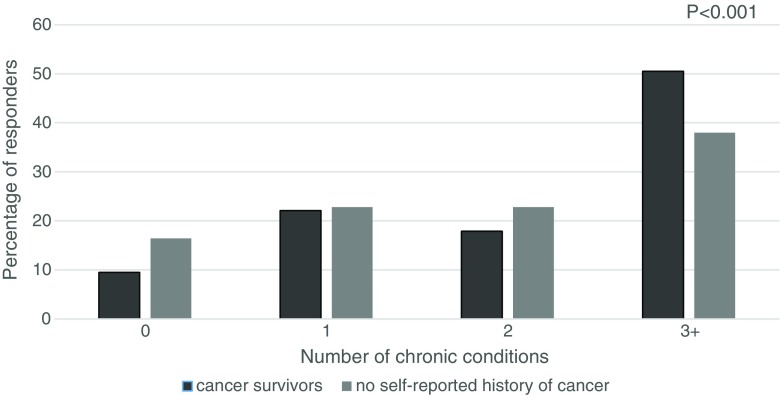
Number of chronic conditions (excluding cancer) according to cancer history. *P* value calculated using chi-squared test of independence

The most prevalent types of cancer among survivors were colorectal (16.6%), prostate (14.2%), and breast (13.9%) comprising about 45% of the reported cancers. In terms of coexisting diseases, three or more chronic diseases were found in over 50% of cancer survivors and in 38% of the non-cancer population (Fig. 1). The mean number of concomitant diseases in cancer survivors was higher than in the non-cancer population (3.8 and 2.3, respectively). Cancer survivors more frequently reported having cardiovascular disorders (75 vs. 69%, *p* = 0.037), endocrine and metabolic disorders (31 vs. 24%, *p* = 0.005), and eye diseases (19 vs. 10%, *p* < 0.001), whereas the prevalence of stroke, chronic diseases of the respiratory or digestive system, kidney diseases, and osteoporosis was similar. Male cancer survivors more often reported having blood disorders than non-cancer men (7 vs. 4%, *p* = 0.03). The analysis for the most prevalent cancers performed separately in men and women demonstrated that the prevalence of osteoporosis (14.6 vs. 5.8%, *p* = 0.02), endocrine disorders (31.7 vs. 17.6%, *p* = 0.02), and renal diseases (10.3 vs. 2.7%. *p* = 0.005) was higher in men with self-reported history of prostate cancer than in men without any cancer history. The distribution of comorbidities in women with breast cancer was comparable to those without the cancer history.

### History of cancer and association with impairments

In the multivariate logistic regression model that controlled for age, sex, marital status, education, and number of comorbidities, cancer survivors were significantly more likely to report falls (OR = 1.38; 95% CI:1.04–1.83), need for assistance (OR = 1.37;95% CI: 1.04–1.81), and poor health status (OR = 1.49; 95% CI:1.08–2.06), compared with subjects without a history of cancer. Cancer history was not associated with depression, cognitive impairment, or impairments in performing IADLs (Table 2).

**Table 2:** Multivariate logistic model identifying factors associated with impairments among PolSenior study participants

	Need for assistance	IADL < 24	GDS ≥ 6 pts	Falls	MMSE < 24 pts	Poor self-reported health status
No cancer history	–	–	–	–	–	–
Cancer survivor	1.37 (1.04–1.81)	0.96 (0.72–1.28)	1.23 (0.93–1.63)	1.38 (1.04–1.83)	0.75 (0.54–1.05)	1.49 (1.08–2.06)
Sex						
Male	–	–	–	–	–	–
Female	1.14 (0.98–1.32)	0.81 (0.69–0.94)	1.21 (1.04–1.41)	1.29 (1.09–1.51)	0.79 (0.67–0.93)	1.07 (0.88–1.30)
Age						
65–79 years	–	–	–	–	–	–
80+ years	5.30 (1.60–6.11)	5.64 (1.90–6.50)	1.46 (1.26–1.68)	2.37 (1.03–2.77)	3.40 (2.93–3.95)	1.60 (1.34–1.92)
Education						
Elementary	–	–	–	–	–	–
University	0.43 (0.33–0.57)	0.19 (0.14–0.24)	0.37 (0.28–0.49)	0.96 (0.73–1.26)	0.15 (0.10–0.21)	0.30 (0.38–0.76)
Marital status						
Not married	–	–	–	–	–	–
Married	0.61 (0.53–0.71)	0.66 (0.57–0.77)	0.66 (0.57–0.78)	0.77 (0.65–0.91)	0.58 (0.49–0.68)	0.94 (0.77–1.15)
No. of chronic diseases						
0	–	–	–	–	–	–
1	1.24 (0.99–1.54)	1.18 (0.95–1.48)	1.16 (0.92–1.47)	1.28 (1.00–1.63)	1.14 (0.91–1.44)	1.45 (1.03–2.05)
2	1.46 (1.17–1.82)	1.06 (0.85–1.31)	1.56 (1.24–1.98)	1.41 (1.10–1.80)	0.93 (0.74–1.17)	1.78 (1.27–2.48)
3+	2.87 (2.33–3.52)	1.77 (1.44–2.17)	2.32 (1.87–2.87)	1.72 (1.37–2.15)	1.02 (0.83–1.26)	3.20 (2.36–4.35)

Data are ORs (odds ratios) and 95% CI (confidence intervals). Dashes indicate reference categories. *IADL* instrumental activities of daily living, *GDS* geriatric depression scale, *MMSE* mini mental state examination

The status of cancer treatment (cured vs. ongoing therapy and less than 5 years since the last treatment) had no significant influence on the prevalence of depression (37.7 vs. 41.6%, *p* = 0.56), need for assistance (45.3 vs. 51%, *p* = 0.38), poor self-reported health status (19.2 vs. 26.1%, *p* = 0.23), deficits in the IADL score (50.4 vs. 54.9%, *p* = 0.49), or falls (32.4 vs. 21.6%, *p* = 0.06), the exception being the declared lower level of social service support in cancer survivors who were cured of cancer (2.2 vs. 10.2%, *p* = 0.008). In the multivariate logistic regression model that controlled for time since diagnosis of cancer, sex, age, education, and number of comorbidities, a functional decline, i.e., decline in physical and cognitive functioning was associated with age; cancer survivors aged 80 years or older had a 5.6-fold higher probability of limitations in the IADL score, a fourfold higher probability of self-reported need for assistance and 3.5-fold higher probability of cognitive disorder compared to cancer survivors aged 65–79 years old. In addition, university education was associated with lower probability of functional decline, as cancer survivors with higher education were less likely to report need for assistance (OR = 0.34; 95% CI: 0.14–0.85) or to have impairments in performing IADLs (OR = 0.13; 95% CI: 0.05–0.35), cognitive impairment (OR = 0.09; 95% CI: 0.02–0.42) and to report poor health status (OR = 0.07; 95% CI: 0.01–0.56). Neither time from cancer diagnosis nor the number of comorbidities was associated with the presence of impairments in cancer survivors. The results are presented in Table 3.

**Table 3:** Multivariate logistic model identifying factors associated with impairments among cancer survivors

	Need for assistance	IADL < 24	GDS ≥ 6 pts	Falls	MMSE < 24 pts	Poor self-reported health status
Time from diagnosis						
< 5 years	–	–	–	–	–	–
≥ 5 years	0.56 (0.29–1.09)	1.06 (0.54–2.12)	0.86 (0.44–1.69)	1.43 (0.70–2.91)	1.09 (0.45–2.64)	0.76 (0.35–1.68)
Sex						
Male	–	–	–	–	–	–
Female	1.07 (0.56–2.04)	0.69 (0.35–1.37)	1.27 (0.66–2.45)	0.68 (0.34–1.34)	0.64 (0.28–1.51)	1.14 (0.51–2.55)
Age						
65–79 years	–	–	–	–	–	–
80+ years	3.99 (2.13–7.45)	5.57 (2.85–10.90)	1.55 (0.83–2.86)	1.79 (0.95–3.39)	3.52 (1.61–7.68)	1.81 (0.85–3.89)
Education						
Elementary	–	–	–	–	–	–
University	0.34 (0.14–0.85)	0.13 (0.05–0.35)	0.39 (0.15–1.00)	0.92 (0.36–2.31)	0.09 (0.02–0.42)	0.07 (0.01–0.56)
Marital status						
Single	–	–	–	–	–	–
Married	1.26 (0.66–2.42)	1.15 (0.58–2.29)	1.58 (0.47–5.31)	0.67 (0.20–2.26)	0.40 (0.09–1.71)	0.88 (0.20–3.89)
No. of chronic diseases						
0	–	–	–	–	–	–
1	1.62 (0.50–5.29)	1.18 (0.33–4.21)	1.58 (0.47–5.31)	0.67 (0.20–2.26)	0.40 (0.09–1.71)	0.88 (0.20–3.89)
2	1.01 (0.30–3.48)	0.92 (0.25–3.34)	1.54 (0.44–5.34)	1.15 (0.34–3.82)	0.33 (0.07–1.59)	0.61 (0.13–2.89)
3+	2.58 (0.86–7.73)	2.11 (0.66–6.81)	1.28 (0.42–3.93)	1.04 (0.36–3.06)	0.70 (0.20–2.46)	1.49 (0.41–5.44)

Data are ORs (odds ratios) and 95% CI (confidence intervals). Dashes indicate reference categories. *IADL* instrumental activities of daily living, *GDS* geriatric depression scale, *MMSE* mini mental state examination

## Discussion

We found that cancer survivors over 65 years of age more frequently experienced falls and presented poor health status in comparison with those without cancer history. Moreover, the octogenarians who survived cancer presented much higher need for assistance due to limitations in functional status in comparison to with younger subjects. Elderly cancer survivors were also more likely to present multi-morbidity than patients without a history of cancer.

Around one third of cancer survivors and less than a quarter of respondents without a cancer history in our study reported having experienced a fall within the previous year. The rate of falls in community-dwelling older adults with cancer varies across studies and is estimated to be 20 to 33% [6]. After controlling for age, sex, education, and comorbidities, a history of cancer was associated with the occurrence of falls in our study. Falls were twice as frequent in elderly men with a history of prostate cancer compared to men without a history of cancer. Exposure to androgen deprivation therapy (ADT) in treating prostate cancer is associated with a higher risk of falls than no such treatment [7]. A study conducted by Mohile et al. also showed that elderly patients with cancer experience a higher prevalence of falls than those without a history of cancer (26.4 vs. 21.9%) [8]. The side effects of cancer therapy, e.g., peripheral neuropathy, fatigue, and pain, are among the factors that could lead to functional impairments and increase the incidence of falls in cancer survivors [9]. Apart from cancer and the long-term effects of treatment, the higher prevalence of falls may also be a consequence of concomitant diseases, other medications, and aging.

Although elderly patients who fall frequently tend to have deficits in functional ability, we did not observe an increased incidence of deficits in performing the IADL tasks in the group of cancer survivors studied. Other studies have also reported maintained independence in cancer survivors. Seventy-one percent of older patients with breast cancer with a median follow-up of 5 years were found to be fully independent in IADL tasks [10]. In contrast, Mohile et al. showed that patients with a history of cancer had a significantly higher prevalence of limitations in IADL tasks than the non-cancer population (49.5 vs. 42.3%) [11]. Furthermore, individuals with cancer reported more limitations in IADLs, with the most common limitations being heavy housework (34%) and shopping (17%) [12]. It is of note that around half of the population in the PolSenior study had some impairment in performing IADL tasks [13]. What is also noteworthy is that in elderly cancer survivors, functional status was found to be associated with the presence of comorbidities and level of education rather than with the presence of cancer or time from diagnosis [14]. In our study, functional decline in cancer survivors was associated with age (5.6-fold higher odds of deficits in performing IADL tasks among patients 80 years and older compared with patients aged 65 to 79 years), but not with the presence of comorbidities or time from diagnosis. Cancer survivors who had university education were less likely to present impairments in IADLs and cognitive dysfunction, to report need for assistance or poor health status than the individuals with elementary education that might suggest that higher education could be protective against detrimental influences of cancer and its treatment or it might contribute to the better capacity to adapt to decreasing functional ability [15]. There were no significant differences in the prevalence of impairments according to the status of cancer treatment. Previous studies have shown an association of cancer history with frailty, and falls are among the common features of frailty [11]. The higher occurrence of falls in our population might be a clinical manifestation of frailty in elderly cancer survivors.

Numerous studies have shown that cancer increases the risk of developing depression in the elderly [16]. Data from the Medicare Current Beneficiary Survey suggested that cancer patients were more likely to experience depression compared with non-cancer controls (OR = 1.15; 95% CI: 1.02–1.30) [8]. In contrast, the occurrence of depression in the PolSenior population of cancer survivors was associated with female sex, age, and number of comorbidities, but not with the cancer diagnosis.

It has been suggested that elderly patients with cancer may have a reduced risk of dementia [17]. These data were supported in a 2015 meta-analysis of three studies analyzing the risk of Alzheimer’s disease in patients with cancer [18]. Also, the results of the Framingham Heart Study of 1278 participants aged 65 years or older without dementia at baseline who experienced cancer indicated that they had a lower risk of Alzheimer’s disease (HR = 0.67; 95% CI: 0.47–0.97) [19]. Only a small number of studies have reported that cancer survivors may experience long-term cognitive deficits. In a study conducted by Heflin et al., 14.5% of cancer survivors had cognitive dysfunction compared with 8.7% of their cancer-free twins [20]. In our study, cancer survivors presented lower prevalence of cognitive impairment in univariate analysis, but it was not confirmed in multivariate logistic regression analysis, as having cognitive impairment was associated with male sex, older age, elementary education, and not being married, but not with cancer survivorship.

The reported incidence of comorbid conditions in cancer survivors varies across studies. Results from a nationwide study among all people living in Denmark indicated a higher prevalence (40%) of a score ≥ 1 on the Charlson Comorbidity Index (CCI) in older cancer survivors than in the non-cancer population (16%). Cancer survivors had 59% higher odds of having a CCI score ≥ 1 (95% CI: 1.57–1.60) after adjustment for age and sex [21]. In a study conducted by Holmes et al. [4], more elderly survivors reported having two or more chronic conditions compared to controls (68 vs. 65%) and the age-adjusted prevalence of cardiovascular diseases (excluding hypertension) was higher in survivors (25 vs. 23%). Three out of four elderly cancer survivors in the PolSenior study reported having cardiovascular disorders. Cancer survivors treated with cardiotoxic therapies are at increased risk of atherosclerosis secondary to inflammation and endothelial dysfunction [22]. We have previously observed endothelial activation in young adult survivors of childhood acute lymphoblastic leukemia early after completing treatment [23]. On the other hand, other studies found a similar prevalence of chronic conditions in elderly cancer survivors and controls, except for a significantly increased frequency of coronary artery disease and emphysema in survivors [24]. In this study, the average number of comorbid conditions in cancer survivors was 3, which was lower than in participants without a history of malignancy. In our study, we found an increased incidence of osteoporosis and endocrine disorders among prostate cancer survivors. The high prevalence (53%) of osteoporosis has been previously documented in men with prostate cancer on ADT [25]. Furthermore, ADT increases the risk of diabetes in older men with prostate cancer, particularly when other comorbidities are present [26]. In addition, ADT may be associated with an increased risk of dementia [27]; nonetheless, we found no increased prevalence of cognitive impairment in men with a self-reported prostate cancer history and according to previously published data, the use of ADT in the studied population was low [28]. Nevertheless, it should be emphasized that due to the cross-sectional nature of the data analyzed in the study, we cannot exclude the hypothesis that the higher number of comorbidities was related to superior health care and a more thorough diagnosis of concomitant diseases in cancer survivors.

Cancer survivorship among the older population has implications for their assessment of their general health status. Our findings indicate that elderly cancer survivors more often report poor health status than individuals without a history of cancer. As mentioned above, the occurrence of falls may contribute to a decline in perceived health status. In a cross-sectional study in older cancer survivors who had a history of falls in the previous year, falls were associated with lower scores for health-related quality of life (HRQOL) and with a prospective decline in HRQOL [29]. In the US National Health Interview Survey in 2010, almost twice as many cancer survivors (47% aged 65 years or older) as adults without a cancer history reported poor mental health-related quality of life, and over a quarter of cancer survivors reported poor physical health-related quality of life. Having more than one comorbidity was found to be associated with poor physical health-related quality of life in both groups [30]. In the PolSenior study, having three or more comorbidities was more strongly associated with poor self-reported health status than cancer survivorship itself.

This study has some limitations. Data were self-reported, which may have resulted in under- or over-reporting of information and patients currently under treatment for cancer might have been underrepresented in the study. Unfortunately, we did not have access to all records of the type and status of cancer treatment. The data on type of cancer, age at diagnosis, and status of cancer treatment could be verified with hospital discharge reports when provided by the patient. Furthermore, cognitive impairment was assessed using screening test (MMSE), which may be influenced by age, education, motor, and visual impairments.

In conclusion, cancer survivors over the age of 65 years experience a higher prevalence of falls, are more likely to report poor health status, and have a higher number of chronic conditions than the non-cancer elderly population, but they maintain independence in IADLs. Advanced age and lower education, but not time from cancer diagnosis, are associated with the occurrence of impairments in older cancer survivors.
